# Genetic diversity in 10 populations of domestic Turkeys by using microsatellites markers

**DOI:** 10.1016/j.psj.2022.102311

**Published:** 2022-11-03

**Authors:** Amado M. Canales, María E. Camacho, Antonio H. Beltrán, Juan V. Delgado, Vincenzo Landi, Amparo M. Martínez

**Affiliations:** ⁎ Department of Genetics, Faculty of Veterinary Sciences, University of Córdoba, 14071, Cordoba, Spain; † Animal Breeding Consulting S.L. Parque Científico Tecnológico de Córdoba , 14071, Córdoba, Spain; ‡ Institute for Agricultural and Fisheries Research and Training (IFAPA), 14004, Córdoba, Spain; § Faculty of Veterinary Medicine and Animal Husbandry, Veracruzana University, 91710, Veracruz, México; # Department of Veterinary Medicine, Università di Bari Aldo Moro, Bari, Italy

**Keywords:** genetic distances, population structure, SSR, evolutionary relationships

## Abstract

The domestic turkey is a native breed in danger of extinction due to the introduction of new breeds specializing in meat production and yield. Turkeys have lost some prominence in urban areas, and only certain breeds of turkeys are preserved in rural areas. Wild and domestic turkeys are different; rural or indigenous turkeys, with black plumage, were domesticated from Mexican turkeys and have been reproduced throughout Latin America. Some of them were taken to Europe in the 16th century and later arrived in North America, where they crossed with another wild species, from which the bronze turkey emerged: the ancestor of all commercial turkeys. The objective of the present work was to evaluate the genetic diversity in 10 populations of domestic turkeys worldwide by using breeds from Europe: Spain and Italy; America: Mexico, United States and Brazil; and the Near East: Iran and Egypt. A total of 522 blood samples of both sexes were collected from domestic turkey populations. Thirty-four microsatellites were used to obtain genetic parameters, and genetic diversity was evaluated. All microsatellites used were polymorphic, and a total of 427 alleles were detected across the 34 markers investigated. In this study, a mean number of 13.44 alleles was found. The four most diverse breeds were from the Andalusia, Mexico, United States, and wild populations, which had the highest mean heterozygosity expected (0.619, 0.612, 0.650, and 0.773) and heterozygosity observed (0.422, 0.521, 0.429, and 0.627), respectively. The MNT348 marker deviated from the HWE in all populations. Our study has shown that the populations close to the species origin are more diverse than those resulting from posterior expansions. Mexican birds were the most diverse, followed by the Spanish populations because Spain imported a large number of turkeys coming from America. Such information can be complementary to other genotypic data required to validate the evolutionary relationships among turkey populations.

## INTRODUCTION

Principal fossil discoveries of turkey ancestors have been found in the south of the United States and north of Mexico (Miller, 1940; Cracraft, 1968); the ancestors of the present turkey *Meleagris gallopavo* (**MG**) evolved from an ancestor that crossed the Straits of Bering when Alaska was connected to Eurasia (Steadman, 2005; Camacho-Escobar et al., 2011). In the Miocene, the real turkeys evolved to the genus *Meleagris (M),* and in the Pleistocene there were at least four species: *M. gallopavo* (current wild turkeys), *M. acellata, M. californica, and M. crassipes*. These last 3 species are extinct (Camacho-Escobar et al., 2011). The genus MG migrated to the southwest of the United States from Mesoamerica, following the route of the great farmlands, and later disseminated in their corresponding areas of distribution (Stark et al., 2010). It has been documented that the domestication of turkeys began in Mexico between 200 and 700 BC (López-Zavala et al., 2008); by pre-Hispanic cultures, the Mayas were the first to domesticate the wild turkey, almost 2000 yr before the Aztecs. The turkey was taken to Europe at the beginning of the 16th century. Cortes mentions that thousands of these birds were raised in the backyard of the palaces of Moctezuma (Camacho-Escobar et al., 2008). From there, the Spanish transported the birds to different parts of America, such as Peru, Chile, and the Antilles.

This is how its mobilization began throughout Europe in the 16th century. Currently, the genus MG is reared in 2 main systems: commercial and backyard, where people raise it freely for self-consumption (Camacho-Escobar et al., 2016). They are found in villages and suburban areas with a low production system and are characterized by poor sanitary practices, factors that adapt to different ecological conditions. Although the turkey varieties are considered a single breed (American Poultry Association, 1926), evidence is emerging about significant biological differences between the populations (Hartman et al., 2006; Lin et al., 2007). The genetic resources currently available from *Meleagris gallopavo* are found in native birds in wildlife and domesticated native species mainly located in Mexico and the United States (Sponenberg et al., 2000). Because of the expansion of industrial poultry production, there is an indiscriminate substitution of creole or native genotypes for improved genotypes (Ceccobelli et al., 2015), which has caused a loss of diversity and provoked crosses of absorption of Creole genotypes by creating improved breeds to generate a larger quantity of meat at a lower price (Dolberg, 2007).

The breeding of domestic turkeys is associated with rural farms, using traditional methods, in rustic facilities that take advantage of the environment of the house and the participation of the family. Conservation of local poultry genetic resources in developing countries should be a priority, as some breeds have sharply deteriorated and even disappeared in recent decades (Cigarroa Vázquez, 2012). FAO defines biodiversity as the genetic variability of different types of animal genetic resources at the level of breeds, species, and genes, from which as many alleles or variants should be kept as possible (FAO, 1996). In this context, the genetics of the turkey is native and constitutes a true genetic background; more than 500 yr after having been exported from Mexico, the genetic constitution of turkeys have been reconformed, originating from different populations in countries where the MG is not native (Mock et al., 2002; López-Zavala et al., 2013). The animal biodiversity includes the diversity of species that are living in a site, its genetic variability and the evolutionary ecological processes that occur at the level of ecosystems and landscapes of which these species interact (Pariacote, 2000). The study of the biodiversity of the MG, its genetic structure and its relationship with other turkey populations is essential to understand its evolutionary potential, health, and sustainability (Chassin-Noria et al., 2005; Rosendo Ponce et al., 2018). Presently, interest in the characterization of native turkey populations has begun, and some studies have been centered on external morphology (Adeyemi and Oseni, 2018) by means of canonical discriminant analysis, but most of the interest has been dedicated to genetic characterization based on DNA markers, mainly microsatellites.

Analysis with microsatellites will provide the molecular information necessary to study the genetic structure and relationship between these populations (Giovambattista et al., 2001, Aranguren-Méndez et al., 2005). Genetic markers are loci that have detectable characteristics that can differ between individuals. Microsatellites have been used in studies of phylogenetic analysis, genetic variability, and differentiation between populations or individuals (Dodgson et al., 1997, Groeneveld et al., 2010, Ceccobelli et al., 2015). From the point of view of improvement and conservation, the principal objective of genetics using molecular microsatellites, is to study, determine and measure the existing variation between and within populations of the same species, to investigate this variation also called polymorphism; originating from spontaneous DNA changes (Aranguren-Méndez et al., 2005). The study of the domestic turkey (MG) has progressed rapidly in the past few years, using molecular biology and genetic markers (Reed et al., 2007) to study the structure of the turkey population outside Mexico and the United States. The populations of domesticated turkeys represent a reservoir of genetic variability and phenotypes that must be explored.

The aim of the present study was to evaluate the genetic diversity in 10 populations of domestic turkeys worldwide by using breeds from Europe: Spain and Italy; America: Mexico, United States, and Brazil; and the Near East: Iran and Egypt. To this end, we used a panel of 34 microsatellites previously tested by our team (Canales Vergara et al., 2020).

## MATERIALS AND METHODS

### Institutional Animal Care and Use Committee Statement

The present research was conducted under the scope of the European Union legislation (2010/63/EU, from the 22 September 2010) and its transposition to the Spanish law document (Royal Decree Law 53/2013). Animals received humane care in compliance with the national guide for the care and the use of laboratory and farm animals in research. As recommended by Royal Decree Law 53/2013, the study protocol was submitted to the legally constituted Ethics Committee of Animal Experimentation of the University of Córdoba, Spain which deemed the study to be exempt from review.

### Sample Collection

Blood samples were collected from domestic turkey populations (local breeds), the animals were not related, and if they were, only the parents and one child were taken. For each individual, blood samples were taken from the brachial vein, placed in 2 mL vials containing ethylenediaminetetraacetic acid (**EDTA**), and stored at −18°C. Additional, blood samples were then collected on filter paper sheets and stored at room temperature until further analysis. Samples were deposited in the Animal Breeding Consulting Laboratory at the University of Córdoba, Spain. Ten local populations of domestic turkeys from different countries and 1 commercial turkey population were included in this study and grouped according to their geographic origin. A total of 548 samples of both sexes from local domestic turkey breeds were available for analysis. From Brazil, 62 samples were collected from rural communities in northeastern Paraiba and Bahia; 51 samples were collected from rural communities in Veracruz, Mexico. Eighty-four samples were from the United States (Bourbon Red, Riley Bronze, Black Spanish, P. Allen Smith, Betsville, Midget White, Quincy Blue, Royal Palm, White Holland). In Africa, 2 breeds were sampled: 47 samples were from Giza Governorate, Egypt, and 36 samples were from rural environments in Iran. Samples from rural environments in Spain were collected, including 130 Andalusian Black from Andalusia (Seville, Cadiz, and Córdoba) and 37 Indiot Mallorquí from the Balearic Archipelago (Mallorca). Sixty-three samples belonging to 2 different breeds were from Italy, with 25 samples from the Parma breed and 38 from the Romagnola breed. Furthermore, 6 wild turkeys in *Eastern Rio* and 4 *Meleagris gallopavo Osceola* were included, and ultimately, 26 samples of commercial turkeys were bred as outgroups.

### Microsatellite Analysis

A total of 34 microsatellite markers (Table 1) for biodiversity studies in turkeys were evaluated, taken from the biodiversity turkey panel by Canales Vergara et al. (2020). Our primer pairs were synthesized by Integrated DNA Technologies, Inc., Coralville, IA or Life Technologies with HPLC purification and labeling was achieved using an M13-tailed primer method (Schuelke, 2000; Boutin-Ganache et al., 2001) that briefly consisted of labeling the forward primers with a specific tail (one for each 4 colors according to ABI instrument filter set G5) and amplification in the presence of 4 complementary FAM, NED, PET, and VIC fluorescently labeled oligos. PCR (polymerase chain reaction) was performed in multiplex reactions and separated in 2 electrophoresis sets in a reaction volume of 10 µL containing 2 µL of Chelex lysate (∼10 ng of genomic DNA), 1X MytaqHS 5X buffer (Bioline GmbH, Luckenwalde, Germany) and 0.5 Unit of MytaqHS Polymerase (Bioline GmbH). The primer concentrations were 0.1 µM for each fluorescent M13 oligo, 0.22 µM for each forward M13-labeled primer and 0.2 µM for each reverse primer. The PCR cycle consisted of a 2-step protocol: 3 min at 95°C for Taq polymerase activation and 35 cycles of 95°C for 30 s, followed by 3 min at the multiplex specific annealing temperature, finally a 60°C final extension step of 20 min for efficient plus A addition. The sizes of the microsatellite alleles were visualized using an ABI prism 3130 XL Genetic Analyzer (Life Technology, Carlsbad, CA) using a POP7 polymer and the internal size standard GeneScan500-LIZ (Life Technology, Carlsbad, CA). Genotypes were read with ABI Genemapper 5 software (Life Technology, Carlsbad, CA).

**Table 1:** Locus studied. Allele number of each locus (NA), effective number of alleles (Ae), mean observed (H_O_) and expected (H_E_) heterozygosity with the standard deviation (SD), polymorphic information content (PIC) and inbreeding coefficient (F_IS_) per locus.

Locus	NA	Ae	H_O_ ± SD	H_E_ ± SD	PIC	*F_IS_*
MGP018	14	5.173	0.477 ± 0.160	0.641 ± 0.137	0.585	0.294
MNT011	16	3.128	0.515 ± 0.244	0.573 ± 0.219	0.511	0.093
MNT013	15	4.797	0.557 ± 0.166	0.632 ± 0.177	0.577	0.116
MNT014	4	2.563	0.301 ± 0.248	0.475 ± 0.236	0.402	0.393
MNT247	21	7.694	0.690 ± 0.148	0.748 ± 0.112	0.700	0.048
MNT258	11	2.554	0.457 ± 0.254	0.542 ± 0.240	0.485	0.194
MNT264	5	1.274	0.199 ± 0.266	0.215 ± 0.239	0.181	0.104
MNT266	23	2.993	0.436 ± 0.150	0.567 ± 0.180	0.498	0.228
MNT274	28	2.618	0.409 ± 0.215	0.531 ± 0.166	0.458	0.145
MNT282	12	2.037	0.428 ± 0.231	0.467 ± 0.248	0.403	0.070
MNT288	6	2.801	0.523 ± 0.199	0.544 ± 0.134	0.470	0.062
MNT294	7	3.705	0.489 ± 0.207	0.555 ± 0.151	0.487	0.077
MNT297	16	2.135	0.474 ± 0.238	0.514 ± 0.227	0.456	0.066
MNT318	7	1.815	0.386 ± 0.213	0.434 ± 0.186	0.372	0.142
MNT331	18	6.779	0.529 ± 0.166	0.675 ± 0.147	0.609	0.161
MNT344	19	4.460	0.543 ± 0.159	0.658 ± 0.130	0.595	0.140
MNT348	14	4.973	0.196 ± 0.103	0.648 ± 0.108	0.578	0.678
MNT353	4	1.569	0.344 ± 0.244	0.358 ± 0.232	0.301	0.017
MNT361	15	5.021	0.618 ± 0.166	0.696 ± 0.136	0.634	0.116
MNT374	11	2.447	0.460 ± 0.158	0.555 ± 0.138	0.479	0.215
MNT386	16	3.553	0.616 ± 0.175	0.619 ± 0.140	0.557	0.020
MNT389	17	2.852	0.463 ± 0.177	0.549 ± 0.176	0.479	0.169
MNT391	14	2.456	0.488 ± 0.258	0.548 ± 0.195	0.464	0.135
MNT393	19	3.912	0.592 ± 0.228	0.631 ± 0.225	0.582	0.037
MNT411	16	4.875	0.574 ± 0.110	0.700 ± 0.124	0.651	0.158
RHT009	12	3.912	0.573 ± 0.139	0.625 ± 0.166	0.561	0.035
RHT024	23	7.389	0.423 ± 0.179	0.710 ± 0.152	0.656	0.378
TUM016	10	1.467	0.185 ± 0.153	0.251 ± 0.230	0.214	0.109
TUM020	30	5.439	0.693 ± 0.121	0.749 ± 0.066	0.691	0.089
TUM023	9	3.317	0.575 ± 0.158	0.584 ± 0.134	0.511	0.025
W075	13	3.072	0.471 ± 0.143	0.560 ± 0.156	0.497	0.147
W077	6	2.530	0.406 ± 0.158	0.503 ± 0.146	0.429	0.185
WT054	12	2.871	0.579 ± 0.198	0.617 ± 0.178	0.555	0.012
WT083	4	2.128	0.419 ± 0.178	0.455 ± 0.108	0.365	0.033
MEAN	13.44	3.454	0.474 ± 0.186	0.563 ± 0.169	0.500	0.143

### Statistical Analyses

Allele frequencies for each locus, mean number of alleles per locus and population (MNA) observed (H_O_) and expected (H_E_) heterozygosity estimates were calculated with Excel microsatellite toolkit 3.11. (Park, 2001). Deviation from Hardy-Weinberg equilibrium (**HWE**) at each locus within populations was tested with Genepop 4.0 software (Rousset, 2008), applying the “Fisher's exact test” and using the “Markov chain algorithm” with default setting. *P* values (Guo and Thompson, 1992) were calculated and corrected for multiple tests using the Bonferroni method (Rice, 1989). FSTAT 2.9.3.2 software (Goudet, 2001) was used to calculate the coefficients for inbreeding within populations (*F_IS_*) and for differentiation among populations (F_ST_) as proposed by Weir and Cockerham (1984) utilizing corresponding *P* values based on 1,000 randomizations. The same software calculated mean allelic richness (R_A_) as a measure of the number of alleles independent of sample size, hence allowing comparison among different sample sizes (Goudet, 2001). Nei´s genetic distances (Nei et al., 1983) between pairs of breeds were estimated, and based on these values, a Neighbor-Net was visualized using the SplitsTree4 Software (Huson and Bryant, 2006). Structure software version 2.2 (Pritchard et al., 2004) was used to cluster individuals based on multilocus genotypes to assess population structures. The analysis involved an admixture model with correlated allele frequencies. One hundred independent runs were carried out with 300,000 iterations during the burn-in phase across the 34 investigated markers, and 600,000 iterations for sampling from 2≤K≥12 (K = number of clusters) to estimate the most likely number of clusters present in the dataset. The average and the standard deviation (**SD**) of the logarithmic likelihood [L(K)] of the data were estimated across 7 runs for each K value. The most plausible number of population clusters was determined by calculating the distribution of the ΔK statistic as described by Evanno et al. (2005). The clustering pattern was visualized using the software Distruct 1.1 (Rosenberg, 2004).

## RESULTS AND DISCUSSION

### Microsatellite Polymorphism

A total of 427 alleles were detected across the 34 markers that were investigated. In this study, a mean number of 13.44 alleles was found. This number was higher than that reported by López-Zavala et al. (2013), who used only 7 markers that showed a mean number of alleles of 9.28. TUM20 exhibited the highest number of observed alleles (30) in all 10 studied local populations. This value is discordant with the results of Burt et al. (2003), in which these markers showed only 7 alleles in the Large White turkey breed. Markers W75 and W77 showed 13 and 6 alleles, respectively, which were higher than those reported by López-Zavala et al. (2013), who used these markers to perform a genetic characterization of domestic and wild turkey populations in Mexico. Their study found 8 and 3 alleles, respectively, whereas MNT14, MNT353, and WT083 showed only 4 alleles in our study (Table 1). Both studies mentioned above were limited in the number of markers and sampling; thus far, our research involves 10 local populations from 7 countries representing 3 continents and 34 markers.

The 34 markers analyzed delivered an H_O_ higher than 0.1 and stretched from 0.170 (TUM016) to 0.690 (MNT247). According to Ott (1992), a locus is polymorphic when H_O_ is greater than 0, equal to 0.1, and highly polymorphic if it is higher than 0.7. Table 1 shows that the average H_O_ (0.474 ± 0.186) exceeds 0.1, indicating that the panel designed for biodiversity in turkeys by Canales Vergara et al. (2020) is polymorphic and extremely efficient in these populations for genetic diversity studies, showing the same accuracy as other more evolved tools such as SNPs. However, until the number of SNPs identified in the turkey genome is significantly increased, microsatellites will continue to be the marker of choice for linkage studies in this species (Smith et al., 2005; Reed et al., 2006).

Previous turkey's studies based on 7 studied microsatellites have reported a mean H_O_ locus of 0.533 in 144 samples (López-Zavala et al., 2008). Smith et al. (2005), using 3 microsatellites, reported an H_O_ of 0.09 in 94 samples in 5 varieties of domestic turkeys in the United States, which shows the variation in the behavior of the used panels. As the references on turkeys are limited, we added some mentions to chickens, where Ceccobelli et al. (2015) in 15 European populations and Granevitze et al. (2007) in 64 populations of chickens estimated H_O_ values of 0.456 and 0.460, respectively, very close to our findings in turkeys. The expected frequencies (H_E_) of heterozygotes (Table 1) per locus ranged from 0.215 (MNT264) to 0.749 (TUM20), and the mean H_E_ was moderate (0.563 ± 0.169), but high compared to previous research in turkeys’ breeds. For example, López-Zavala et al. (2013) reported a value of 0.560 in turkeys and 0.520 in chickens, as presented by Granevitze et al. (2007), but lower than native chickens from Korea: 0.630 (Kong et al., 2006).

The polymorphic information content (**PIC**) of the investigated single *loci* ranged from 0.181 (MNT264) to 0.691 (TUM20). Markers showing values higher than 0.50 were considered very informative, markers with values between 0.25 and 0.50 were considered mildly informative and markers falling below 0.25 were fairly informative (Serrote et al., 2020). In this study, 47% of the microsatellites can be considered, based on the PIC value, as very informative, while 44.11% are mildly informative and only 8.82% were highly informative.

A broad variation was found for the *F_IS_* index value among loci, ranging from 0.012 (WT054 locus) to 0.678 (MNT348 locus). The overall *F_IS_* value among all loci was significantly higher than zero (0.143), and the Ae varied between 7.694 (MNT247) and 1.274 (MNT264), with an average of 3.454 effective alleles in the 34 studied microsatellites. The *F_IS_* and the Ae indicated a certain level of heterozygote deficiency; all 34 examined microsatellite markers contributed to this observed heterozygote, this may be due the fact that the samples are composes of individual from different populations.

### Within Breeds Study

The mean values of the number of alleles per population, expected and observed heterozygosity, allelic richness, private allelic richness, the number of loci deviating from HWE in each population, and inbreeding coefficients per breed are presented in Table 2. The observed heterozygosity indicates how heterogeneous the analyzed populations are; the expected and observed heterozygote frequencies within populations across loci were 0.570 (ranging from 0.484 to 0.773) and 0.468 (ranging from 0.352 to 0.627), respectively. The 4 most diverse breeds were AND, MEX, USA, and WIL, which had the highest mean H_E_ (0.619, 0.612, 0.650 and 0.773) and H_O_ (0.422, 0.521, 0.429 and 0.627), respectively. The population of BRA presented moderately low heterozygosity in comparison with the average, which means that these populations can be under reproductive management. A low rate of heterozygosity was present in populations of Bergische Kraeher H_O_ 0.320 and H_E_ 0.440 chickens (Granevitze et al., 2007), New Hampshire Red H_O_ 0.295 and H_E_ 0.290 chickens (Tadano et al., 2007), and Sobrarbe H_O_ 0.370 and H_E_ 0.500 chickens (Ceccobelli et al., 2015). The MEX and WIL populations showed high values of H_O_ 0.532 and 0.627, respectively, and H_E_ 0.612 and 0.773, respectively. Similar values were delivered in previous investigations in domestic turkey populations in Mexico, where H_O_ and H_E_ were 0.535 and 0.606, respectively (López-Zavala et al., 2013). All these turkey studies were developed with a small panel of microsatellites, so the findings must be considered preliminary reports. Only our results could be compared with chicken research at the same level because our design completely fitted the international recommendations for diversity studies (ISAG,FAO (1996).

**Table 2:** Turkey breeds studied. Acronym (ID), country of origin and sample size (N) of each breed. Mean observed (H_O_) and expected (H_E_) heterozygosity with the standard deviations (SD). Mean number of observed alleles (MNA). Mean allelic richness per locus corrected for sample size breed (R_A_). Number of loci deviated from Hardy-Weinberg equilibrium per breed (dHWE) and inbreeding coefficient (*F_IS_*) per breed.

Population	ID	Country	N	H_E_ (SD)	H_0_ (SD)	MNA (SD)	R_A_	dHWE	*F_IS_* (C.I 95%)
MEXICAN	MEX	MEXICO	51	0.612 (0.030)	0.521(0.012)	6.76 (3.52)	2.93	3	0.150 (0.09316–0.18579)
BRASILIAN	BRA	BRAZIL	62	0.484 (0.037)	0.352 (0.010)	4.50 (1.97)	2.35	10	0.274 (0.21554–0.31530)
USA	USA	USA	84	0.650 (0.027)	0.429 (0.009)	8.35 (3.17)	3.10	24	0.345 (0.30359–0.37554)
WILD TURKEY	WIL	USA	6	0.773 (0.029)	0.627 (0.036)	5.09 (2.01)	3.78	0	0.209 (−0.14187 to 0.20798)
ANDALUSIAN BLACK	AND	SPAIN	130	0.619 (0.026)	0.422 (0.007)	6.50 (2.70)	2.87	29	0.318 (0.28784–0.34324)
INDIOT MALLORQUÍ	MAL	SPAIN	37	0.511 (0.035)	0.442 (0.014)	3.82 (1.68)	2.41	8	0.136 (0.06621–0.17601)
PARMA	PAR	ITALY	25	0.503 (0.026)	0.494 (0.017)	3.26 (1.16)	2.29	2	0.020 (−0.09640 to 0.08749)
ROMAGNOLO	ROM	ITALY	38	0.524 (0.026)	0.475 (0.013)	4.06 (1.69)	2.47	5	0.095 (0.02727–0.13475)
EGYPTIAN	EGY	EGYPT	47	0.518 (0.035)	0.454 (0.012)	3.76 (1.56)	2.42	5	0.124 (0.06872–0.15508)
IRANIAN	IRN	IRAN	36	0.507 (0.034)	0.468 (0.015)	3.68 (1.70)	2.36	3	0.078 (0.01499–0.10539)
COMMERCIAL	COM	COM	26	0.488 (0.038)	0.523 (0.016)	4.06 (1.98)	3.73	8	0.072 (−0.21764 to 0.01236)
MEAN			548		0.563 ± 0.032	0.473 ± 0.015	4.90 ± 2.10	2.79 ± 0.547	

Regardless, our study has shown that populations close to the species origin are more diverse than those resulting from posterior expansions. Mexican birds were the most diverse, followed by the Spanish populations, as Spain is a receptive country of the turkeys coming from America.

The MNA per population ranges from 3.68 (1.70 SD) in the IRN to 8.35 (3.17 SD) in the United States and has an average value of 4.97 ± 1.68 alleles per locus in the breeds. The turkey population from the United States presented a higher number of alleles per locus in comparison to all populations under analysis, perhaps because it includes a wide variety of animals and populations from very diverse areas of the region and is a very polymorphic population. Second, the population from the MEX and AND populations showed the lowest MNA compared to the average population, and the populations from the PAR, IRN, and EGY were less polymorphic. This is a common approach for evaluating difference in genetic management among populations, while the United States, Mexican, and Andalusian populations point out open breeding without intensive selection, the other studied populations showed different levels of inbreeding management.

R_A,_ from an evolutionary point of view, is an important parameter indicative of genetic variability and diversity. This fact is determined by the initial number of alleles R_A_ means values (2.69 ± 0.474) varying within a range, from 2.35 BRA to 3.78 in WILD, assuming a minimum sample size of 20 heterozygous individuals. According to this parameter, WIL, USA, and MEX populations presented a high degree of allelic richness, and these results show the evolutionary potential of adaptability of the populations (Greenbaum et al., 2014; Ceccobelli et al., 2015; Vargas et al., 2015). The Hardy-Weinberg equilibrium (**HWE**), following Bonferroni correction, showed that AND was the population with 29 markers of 34 microsatellites deviated from each other (Supplementary Table 1). This could be because this population is distributed throughout a wide territory and probably has some internal structure (Garnier-Géré and Chikhi, 2013), which will be tested in the structure analysis presented later on. Some populations presented only 2 of 34 loci in disequilibrium, such as PAR (see Supplementary Table 1). The MNT348 marker deviated from the HWE in all populations, which is possible due to some linkage to functional or adaptive genes.

Deviations are expected in populations substructured in flocks, within populations that are isolated from each other, or if inbreeding has occurred in populations as a whole. It was in correlation with the *F_IS_*_,_ which was significantly different from zero. This positive association explains the deficiency of heterozygotes in those populations that are in HWE imbalance; however, it is possible that other factors independent of those previously described also affect the deviation (Woelders et al., 2006; Granevitze et al., 2007; Blackburn et al., 2011,). For example, it is probable that some of the studied populations are substructured, such as the case of AND as pointed out before, which violates the basic assumptions of HWE, including random sampling in some of them (Wittke-Thompson et al., 2005). The populations studied in the present research were sampled as described in Table 2; however, this deviation of substructures cannot be proven for populations because there are populations that do not have many loci in HWE deviation. The coefficient of inbreeding (*F_IS_*) per population was significant in all the populations studied except in WILD and PAR turkeys. The rest of the populations presented significant values of *F_IS_* with AND and USA being the populations that showed the highest values due to the substructures that exist in each of the 2 populations. These levels of *F_IS_* need to be controlled because they can present several problems, such as a high incidence of recessive genetic diseases in the future. In other reports, lower and higher *F_IS_* were found, as the characterization of domestic turkey populations in Mexico with 0.023 in domestic and 0.266 in wild populations (López-Zavala et al., 2013) and in other animals such as hens, data as 0.089 (Ceccobelli et al., 2015), 0.090 (Granevitze et al., 2007), and 0.109 (Tadano et al., 2007).

### Among Breeds’ Study

Table 3 shows the genetic differentiation index between pairs of populations (*F_ST_*). Values ranged from 0.078 (AND vs. MEX) to 0.307 (WIL vs. BRA). We integrated the commercial turkey outgroup population to observe the population that was closest to the commercial breeds, and the result was that the nearest population was the USA. This could be explained because the breeds from the USA are the base of the commercial turkeys, whereas the highest genetic differentiation was between the USA and WIL. Previous investigations in domestic and wild animals in Mexican populations showed lower results of *F_ST_* 0.167 (López-Zavala et al., 2013). In a general description, the populations with high genetic differentiation were the USA, MEX, and AND populations. This result supported the hypothesis of decreasing diversity as the number of species increase globally, higher in the origin and lower in newly formed populations, probably due to sequential bottlenecks that occurred in the historical dissemination of the species. Among these 3 populations, moderate genetic differentiation was observed, probably because they share common ancestors. The low *F_ST_* between these 3 populations could be explained because the focus of domestication of *Meleagris gallopavo* occurred in Mexico.

**Table 3:** Pairwise *F_ST_* between the studied populations below the diagonal and genetic distance D_A_ of Nei over the diagonal. Acronym (ID), MEX (Mexico), BRA (Brazil), USA (United States), WIL (wild turkey), AND (Andalusia), MAL (Indiot Mallorquin), PAR (Parma), ROM (Romagnolo), EGY (Egypt), IRN (Iran), and COM (Commercial).

ID	MEX	BRA	USA	WIL	AND	MAL	PAR	ROM	EGY	IRN	COM
MEX	0	0.282	0.207	0.522	0.173	0.251	0.254	0.232	0.225	0.296	0.309
BRZ	0.199	0	0.237	0.584	0.226	0.244	0.308	0.250	0.222	0.263	0.272
USA	0.096	0.157	0	0.455	0.151	0.196	0.262	0.233	0.161	0.242	0.239
WIL	0.189	0.307	0.161	0	0.510	0.531	0.589	0.575	0.553	0.594	0.587
AND	0.078	0.165	0.074	0.193	0	0.143	0.190	0.164	0.175	0.223	0.237
MAL	0.155	0.229	0.102	0.262	0.101	0	0.252	0.237	0.210	0.235	0.286
PAR	0.166	0.283	0.167	0.277	0.121	0.219	0	0.221	0.227	0.266	0.315
ROM	0.138	0.229	0.162	0.279	0.110	0.227	0.193	0	0.225	0.263	0.308
EGY	0.123	0.164	0.080	0.253	0.096	0.167	0.184	0.166	0	0.277	0.281
IRN	0.171	0.223	0.117	0.298	0.123	0.174	0.216	0.233	0.192	0	0.262
COM	0.196	0.239	0.151	0.301	0.166	0.257	0.255	0.238	0.218	0.203	0

The European conquerors brought the turkey to Europe in the 17th century to Spain and distributed them in Europe, and later, the British reintroduced them, where now it is the southwest of the United States and the American indigenous communities used turkeys as domestic birds (Cracraft, 1968; Lin, 2005; Camacho-Escobar et al., 2016). The description in Table 3 shows that all populations except those described previously were highly genetically differentiated. This can be corroborated with the *F_ST_* distances (Table 3) and the Neighbor-Net dendogram presented inFigure 2.

**Figure 1:**
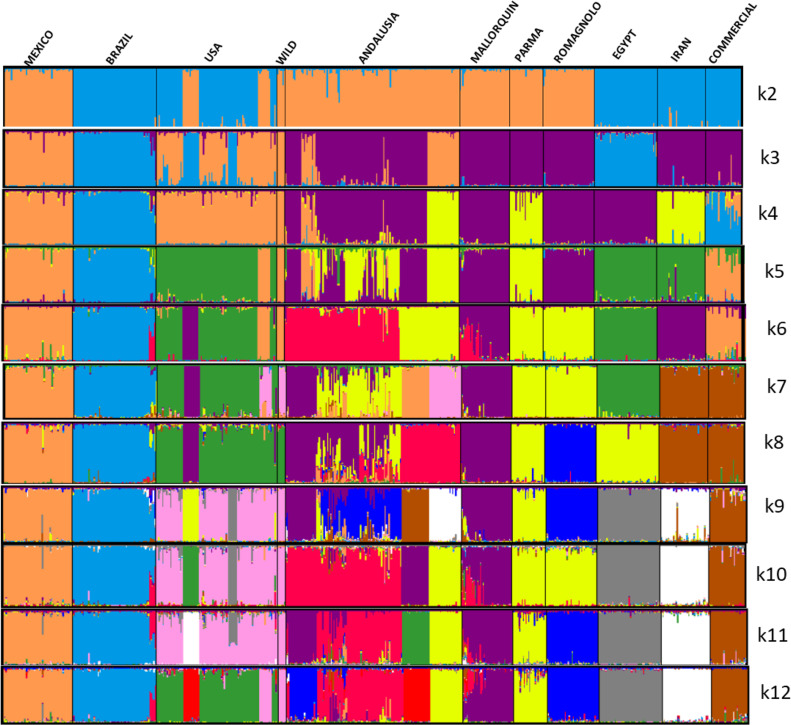
Graphical representation of the estimated membership fractions of individuals of the populations analyzed in each of the K inferred clusters, K = 2 to K = 12 (Burn-in 3,000,000; interactions: 1,000,000).

**Figure 2:**
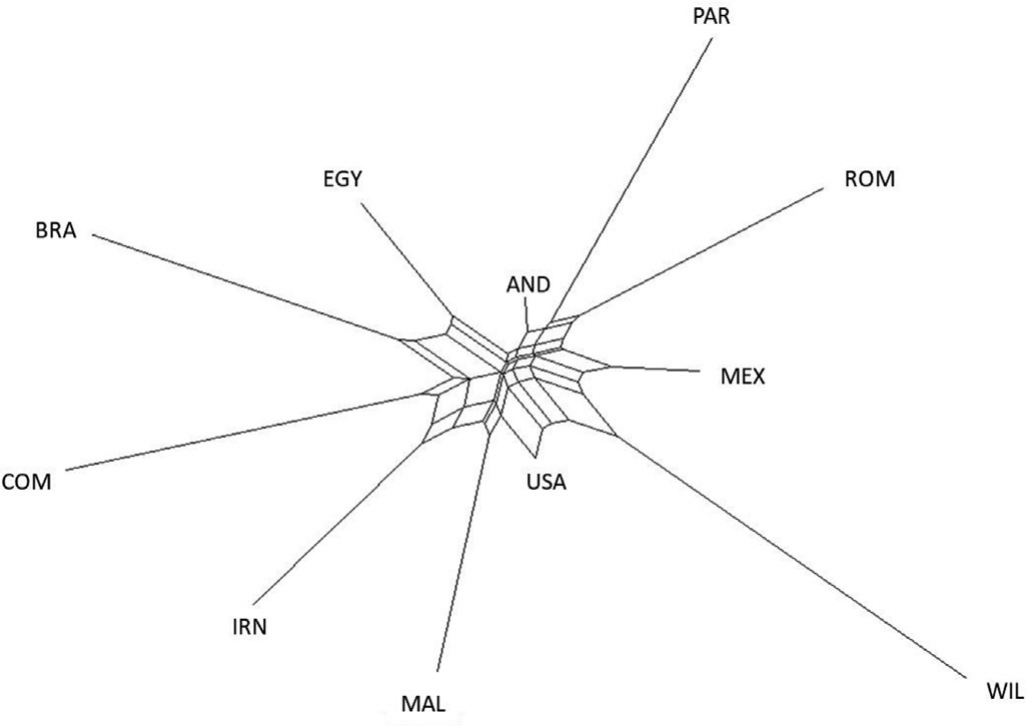
Neighbor-Net dendogram obtained by the genetic distance of the Nei distance. Populations MEX (Mexico), BRA (Brazil), USA (United States), WIL (Wild turkey), AND (Andalusia), MAL (Indiot Mallorquin), PAR (Parma), ROM (Romagnolo), EGY (Egypt), IRN (Iran), and COM (Commercial).

D_A_ genetic distances between each pair for all eleven turkey populations, based on 34 microsatellite loci genotypes, are shown in Table 3. The genetic distance ranged from 0.143 (AND vs. MAL) to 0.594 (IRN vs. WIL). The shortest distance from the commercial turkeys was in the United States (0.239), and the highest was in the WIL population. It is not surprising to note that the genetic distance between the four populations from Europe (AND, MAL, PAR, and ROM) was found to be quite low, highlighting the short distance to the AND population with the USA (0.151) and MEX (0.173). This short distance is because once the turkey was introduced in Spain from the New World and later in Europe (Dickson, 1992), it spread throughout the continent, forming local groupings, of which the previously mentioned populations are ancestors of these breeds (López-Zavala et al., 2008).

The Neighbor-Net dendrogram in Figure 2 indicates a close genetic relationship between populations from the same country regions, such as PAR and ROM that share the same cluster. The other 9 populations were separated into 3 clusters: MEX-WIL-USA. These 3 populations have a minimum distance, sharing the same cluster and demonstrating some common genetic basis in the domestication event. MAL and AND formed individual branches, and MAL was closer to the American populations. AND was in the middle of EGY and PAR-ROM but closer genetically to the Italian populations. Turkey from Iran, Brazil, and Egypt shared the same cluster, which includes the commercial population. This could be explained by the influence of the COM in the rest of the populations of the cluster, as can be corroborated in the Structure results. *M. gallopavo* was introduced in Iran from Europe and increasingly spread in some provinces, such as Khorasan, Gilan, Mazandaran, Western and Eastern Azerbaijan, Markazi, Fars, Esfahan, and Kerman. In these provinces, these birds are traditionally grown by farmers (Rassouli et al., 2016).

Regardless, the geographical distribution of the populations is clear. The populations from America and Wild populations are the closest. Additionally, European populations are closely related, especially MAL, and both Italian populations showed Mediterranean neighboring. Finally, both Near Eastern breeds are also relatively close. Some slight influences of the commercial breeds (COM) over some of the studied breeds were also evident. With breeds from the USA being the original breed and different breeds in other continents because migration.

Bayesian clustering methods allow for the assignment of individuals to groups based on their genetic similarity and provide information about the number of ancestral populations underlying the observed genetic diversity. The results of this analysis indicate for the 11 populations analyzed that the most likely number of ancestral populations is k11, suggesting that the most significant division was by population or by groups of closely related breeds. When studying K2, two groups were observed: MEX with the WIL and from European populations, and the other groups conformed with BRA, USA, EGY, IRN, and COM. From K3 to K9, it is possible to observe that all the populations are integrated and separate, and only the populations of AND and USA present a significant substructure in K7. This can be explained in the USA populations because the samples are from recognized breeds created by means of inbreeding reproduction and in the AND because of the existence of internal substructure due to the different varieties of turkey that are present in the territory. From K10 to K11, all breeds clustered independently when 11 populations were considered (the most likely K value). It was expected that Andalusia to be grouped as a single population, because samples were collected from phenotypically similar turkeys that conformed to the Andalusian population standards, but it was not observed. These results could be a sign of internal structure produced by genetic drift but also because exotic introgressions put the population at risk of extinction. For this reason, urgent measures must be applied to avoid the destruction of the local Andalusian turkey populations.

## CONCLUSIONS

We can consider that the 34 markers are sufficiently polymorphic to be used in a genetic characterization study and are recommendable in genetic diversity studies in the turkey species, the information obtained in this research will be useful for the purposes of conservation and better management of traditional production systems.

The wild turkey population showed an impressive level of polymorphism, which is a sign of high genetic diversity and a good state of conservation.

A direct genetic relationship among the domestic turkey populations first domesticated from México and the United States and the wild ancestors was evidenced.

The south of Spain (Andalusia), as the first point of contact of turkeys in Europe, still maintains strong genetic links with the original populations from México and the United States, even showing some internal structure due to genetic drift and recent introgressions. This short distance between these 3 populations gives us an idea and leaves a fingerprint of where these populations come from and how they have been migrating around the world.

All the studied populations showed a clear genetic definition supporting their official recognition as independent breeds in the same terms as in other domestic species.

There is some evidence that these local breeds already have admixtures with commercial breeds, but it is still possible to rescue them of the extinction, such as the case of the Andalusian and Egyptian breeds. However, in general, we found that the local breed is still genetically distant from the influence of commercial breeds.

In the studied local breeds, it is possible to use the genetic profiles defined in the present study to identify which breed the individual belongs to; such information can be complementary to other genotypic data required to validate the evolutionary relationships among turkey populations.

## Appendix Supplementary materials

Image, application 1
