# Supplementary material for: Genetic diversity in 10 populations of domestic Turkeys by using microsatellites markers
Source: Poult Sci. 2022 Nov 3;102(1):102311. doi: 10.1016/j.psj.2022.102311 (PMC9758563; doi:10.1016/j.psj.2022.102311)
Supplement: Supplementary file 1 [file mmc1.docx]

Supplementary table 1.- Hardy Weinberg disequilibrium P value significance for each population. *P<0.001

|  | MEX | BRA | USA | WIL | AND | MAL | PAR | ROM | EGY | IRN | COM | Total |
| --- | --- | --- | --- | --- | --- | --- | --- | --- | --- | --- | --- | --- |
| MGP018 |  | * | * |  | * | * | * |  | * |  |  | 6 |
| MNT011 |  | * | * |  | * |  |  |  |  |  |  | 3 |
| MNT013 |  |  | * |  | * |  |  |  |  |  |  | 2 |
| MNT014 | * | * | * |  | * | * |  | * | * |  |  | 7 |
| MNT247 |  | * | * |  | * |  |  |  |  |  | * | 4 |
| MNT258 |  |  |  |  | * |  |  | * |  |  |  | 2 |
| MNT264 |  |  |  |  | * | * |  |  |  |  | * | 3 |
| MNT266 |  | * | * |  | * |  |  |  |  |  |  | 3 |
| MNT274 | * |  |  |  | * |  |  |  |  |  |  | 2 |
| MNT282 |  |  |  |  | * |  |  |  |  |  |  | 1 |
| MNT288 |  |  |  |  | * |  |  |  |  |  |  | 1 |
| MNT294 |  |  | * |  | * |  |  |  |  |  |  | 2 |
| MNT297 |  |  | * |  |  | * |  |  |  |  |  | 2 |
| MNT318 |  |  | * |  | * |  |  |  |  |  |  | 2 |
| MNT331 | * | * | * |  | * |  |  |  |  |  |  | 4 |
| MNT344 |  |  | * |  | * |  |  |  |  |  | * | 3 |
| MNT348 |  |  | * |  | * | * | * | * | * | * | * | 8 |
| MNT353 |  | * | * |  |  |  |  |  | * |  |  | 3 |
| MNT361 |  | * | * |  | * |  |  |  |  |  |  | 3 |
| MNT374 |  |  | * |  | * |  |  |  |  |  |  | 2 |
| MNT386 |  | * | * |  | * |  |  |  |  |  |  | 3 |
| MNT389 |  | * | * |  | * |  |  | * |  |  |  | 4 |
| MNT391 |  |  | * |  | * |  |  | * |  |  |  | 3 |
| MNT393 |  |  |  |  | * |  |  |  |  |  | * | 2 |
| MNT411 |  |  | * |  | * | * |  |  |  |  |  | 3 |
| RHT009 |  |  | * |  |  |  |  |  |  |  |  | 1 |
| RHT024 |  |  | * |  | * | * |  |  |  |  | * | 4 |
| TUM016 |  |  |  |  | * |  |  |  | * | * | * | 4 |
| TUM020 |  |  | * |  | * |  |  |  |  |  | * | 3 |
| TUM023 |  |  | * |  |  |  |  |  |  |  |  | 1 |
| W075 |  |  |  |  | * | * |  |  |  | * |  | 3 |
| W077 |  |  |  |  | * |  |  |  |  |  |  | 1 |
| WT054 |  |  |  |  | * |  |  |  |  |  |  | 1 |
| WT083 |  |  | * |  |  |  |  |  |  |  |  | 1 |
